# Bioeconomy and Circular Economy: Implications for Economic Evaluation in the Post-COVID Era

**DOI:** 10.1007/s43615-021-00113-1

**Published:** 2021-11-18

**Authors:** Davide Viaggi, Matteo Zavalloni

**Affiliations:** Department of Agricultural and Food Sciences, University of Bologna, Viale Fanin, 50, 40127 Bologna, Italy

**Keywords:** Bioeconomy, Circular Economy, Post-COVID era

## Abstract

The objective of this paper is to review selected insights about the current economic research on the Bioeconomy and circular economy, with a particular focus for the role of primary sector, and to derive implications for organisation, evaluation and valuation practice in the context of the post-COVID era. A framework for the analysis of optimal level of circularity and related economic and evaluation concepts is developed for this purpose. We highlight how higher focus on circularity will increase the complexity of market relationships, contributing to flexibility, but also to uncertainty. The paper argues that these issues will become more important in the post-COVID era, due to the plea for increasing Bioeconomy resilience. New organisational concepts and models are hence needed. Evaluation, on the other hand, will need to be embedded even more in the decision-making processes, in spite of the increasing uncertainty and difficulties in evaluation.

## Introduction and Objectives

The concept of Bioeconomy has developed in the last 15 years and is growingly adopted to define the aggregate of sectors using biological resources. The Bioeconomy is actually defined in different ways in different contexts, in particular because Bioeconomy strategies are focusing on different sectors depending on countries and areas of the world.

In the EU, a new boost to the Bioeconomy has been given by the launch of the revised Bioeconomy strategy by the EU Commission in October 2018 [11]. According to the strategy, the Bioeconomy is defined in a comprehensive way as follows: “The bioeconomy covers all sectors and systems that rely on biological resources (animals, plants, micro-organisms and derived biomass, including organic waste), their functions and principles. It includes and interlinks: land and marine ecosystems and the services they provide; all primary production sectors that use and produce biological resources (agriculture, forestry, fisheries and aquaculture); and all economic and industrial sectors that use biological resources and processes to produce food, feed, bio-based products, energy and services. To be successful, the European Bioeconomy needs to have sustainability and circularity at its heart. This will drive the renewal of our industries, the modernisation of our primary production systems, the protection of the environment and will enhance biodiversity” [11].

The economic and business literature is working to identify structuring concepts allowing a better understanding of this idea. A broad review is provided by Viaggi [25], while recent updates in terms of economic organisation are illustrated in Viaggi [26]. Besides the sector coverage, key qualifying features of the Bioeconomy are that, on the one hand, it builds on a wide range of modern technologies; on the other hand, it aims at sustainability, by explicitly including ecosystem management.

In recent years, the concept of Circular Economy (CE) has also become mainstream to answer the societal concern for resources limitations and planet boundaries. As a result, the focus of policy and research is today moving towards the concept of Circular Bioeconomy (CBE). It is usually acknowledged that the Bioeconomy does not imply circularity, which means that a circular Bioeconomy needs to be achieved through a deliberate policy strategy backed by supporting research. In this process, understanding the economic features of a CBE is important, as well as providing suitable evaluation and valuation practices to support decision-making.

In this direction, two gaps emerge in the literature. First, in spite of the growing attention being taken by circularity, the development of a rigorous economic framework to interpret its role in the Bioeconomy is still absent in the literature. Second, circularity is not a property that can be understood in isolation, but rather requires to be framed into a number of other organisational properties of the Bioeconomy systems, such as separability of processes, cascading and flexibility. The need to clarify these issues interacts with the re-positioning of the Bioeconomy in the post-COVID era. In particular the trade-offs between short-term efficiency (required for competitiveness), flexibility and resilience (advocated for crisis management) and circularity (advocated to ensure longer-term resource efficiency) may become more evident in the post-COVID context.

The objective of this paper is to review selected insights about the current economic research on the Bioeconomy and CE, with a particular focus on the role of the primary sector, and to derive implications for organisation, evaluation and valuation practice in the context of the post-COVID era through the proposal of a conceptual economic framework of the CBE.

The reminder of the paper is organised as follows. In the “Main Conceptual, Technological and Organisational Issues in the Bioeconomy” section, we review selected concepts shaping the organisation of Bioeconomy systems. In the “Bioeconomy and Circular Economy” section. we review the main concepts linking the Bioeconomy and the CE (in particular CE application to the Bioeconomy). Based on the integration of these two sections, in the “Towards a Framework for Circular Bioeconomy Organisation and Evaluation in the Post-COVID Era” section, we provide a framework for the analysis of circular Bioeconomy, focusing in particular on market and organisation, evaluation and valuation practice in the context of the post-COVID era. The “Discussion and Final Remarks” section concludes with some discussion and final remarks.

The literature review part of this paper does not follow a systematic methodology. The literature was selected after a search in Scopus using the keywords “CIRCULAR” AND “BIOECONOMY” and filtering for papers in “Business, management and accounting” and “Economics, econometrics and finance”. This yielded 92 papers. Upon inspection of the papers, however, we found that very few of them addressed the problem of framing and modelling the circular Bioeconomy. So we proceeded working on few selected papers and using them to enrich existing frameworks for the Bioeconomy. The outcome is presented in the “Towards a Framework for Circular Bioeconomy Organisation and Evaluation in the Post-COVID Era” section, and it is the main result of this work.

## Main Conceptual, Technological and Organisational Issues in the Bioeconomy

Before addressing the issue of circularity, we review the main issues affecting the economic organisation of the Bioeconomy and potentially interacting with circularity. This section is not intended to provide a thorough literature review on the topic, but rather to present selected topics building on already established literature, focusing on organisational issues drawn from Chapters 4, 5 and 6 of Viaggi [25].

While there are many aspects of the Bioeconomy that can be relevant for the objective of this paper, in this section, we focus on six interconnected issues, roughly organised in the terms of increasing scale:

- The relevance of innovation processes
- The increased technological separability of processes
- The importance of flexibility and cascading
- Consumer’s acceptability and market segmentation
- The (partly consequent) increase in the complexity of the value chains
- The growing relevance of system-level organisational concepts

The relevance of innovation for the Bioeconomy is paramount [19, 23]. Most of the concrete policies active in the Bioeconomy sector are based on the promotion of research and innovation [8], or regulating property rights on new technologies. Innovation implies that technical relationships change rapidly over time, which reflects also in high variability of costs and profits. As a result, also uncertainty is very high. In addition, profits are highly linked to the ability to market innovation properly, and, in turn, values are highly attached to expectation and information. Innovation is not only important in changing input-output relationship, but also in affecting the separability points and the flexibility of the processes, hence having effects on all the following points in this section. Finally, innovation may translate in completely new products with an issue in establishing consumers’ preferences on these products.

**Fig. 1:**
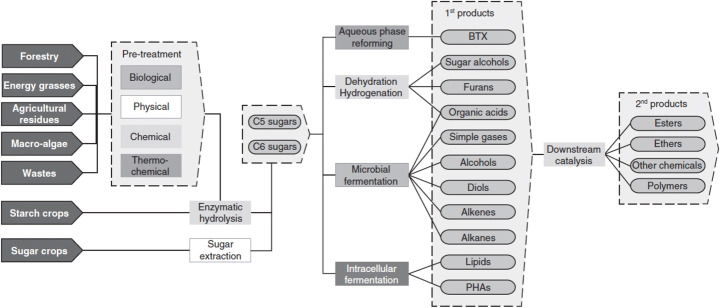
Example of platform chemicals in the sugar sector. Source: Taylor et al. [24]

Many technologies in the Bioeconomy however are more interesting for their separability implications. One of the main interesting strategies of the Bioeconomy is indeed in the process of breaking down biomass into very simple components and in the ability of recomposing them into a variety of compounds and final products. The study by Taylor et al. [24] is paradigmatic in this direction, showing how a wide variety of biomass sources can be used to generate two types of sugar molecules, that can then be used to obtain complex materials (Figure 1). The increased separability of processes emphasises a direction of change of technology that is already under way, allowing higher specialisation of production and also higher complexity of the value chains.

However, separability itself is not telling the whole story of Bioeconomy organisation. On the one hand, flexibility is a characteristic of growing importance especially at processing plant level. Flexibility in this context means the ability of a plant to work with different feedstocks, adapting to availability and costs. It may also mean for a processing plant to be able to process different types of products. This is also explicitly bringing to the emergence of the concepts of flexible biomass and flexible processing plants [10, 29]. On the other hand, the current development of the Bioeconomy is dominated by the idea of a cascading approach, incorporated in the biorefinery concept [4]. The cascading approach implies in particular the ordered treatment of biomass to the extraction of most valuable compounds, down to the production of bioenergy, hence ensuring a complete and efficient use of biomasses available [16].

In this new context, consumers and consumptions decisions have clearly a paramount role, and this is connected to the mechanisms of preferences building, that is in turn connected to information [15]. Many Bioeconomy technologies are known for the debate they have brought concerning acceptability. The most well-known example is probably genetic modified organisms. The fact that many products of the Bioeconomy may bring process innovations with uncertain acceptability reflects in high instability of preferences and market, and an important role of information. Even more important, the growing variety of products with rather different acceptability properties and appreciation by different consumers is leading to an exponential segmentation of markets and consumers groups [9]. This implies not only acknowledging, but also exploiting or even encouraging differentiation of behaviour and consumption habits.

**Fig. 2:**
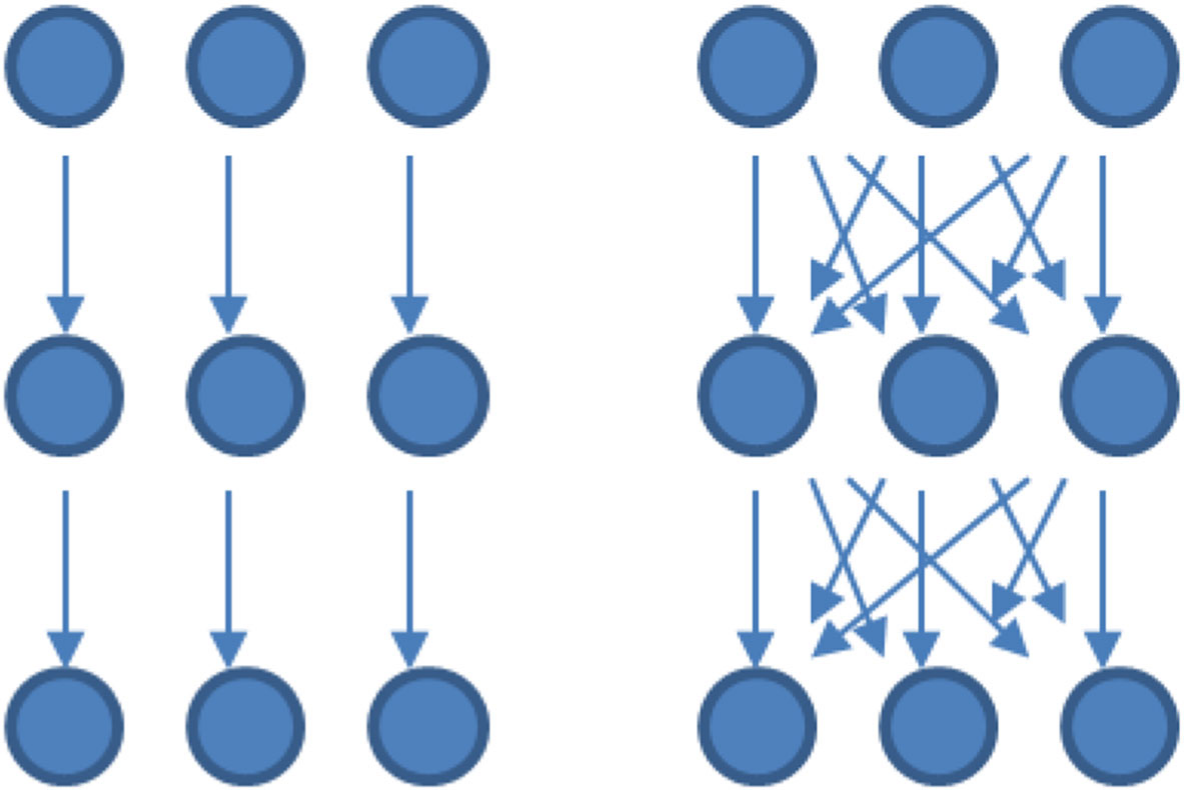
Evolution from value chains to a web of relationships. Source: own figure

As an effect of the above, the evolution of value chains becomes important and goes in the direction of increasing interaction between different value chains and towards the need to go beyond the value chain concept itself, towards representing the Bioeconomy system rather as a web of relationships, with a much higher number of interactions (Figure 2). Here the concept of value web seems a promising pathway to go beyond that of value chain, which is somehow insufficient to account for the increased degree of interaction [22, 27]. This process is also functional to a better resilience of the system. From an economic point of view, it is clear that there are trade-offs with potential higher coordination costs and, in general, transaction costs.

As for the primary sector, the transformations above imply a much higher substitutability of biomass that would be further emphasised by CE (see below). At the same time, however, the distinction between cultivated and natural environment is fading. Cultivation is growing (see forestry, aquaculture) and substituting pure harvesting (e.g. fishing). Humanity is taking a number of actions to manage the functioning of the ecosphere (one could now say that we are approaching a level where we “cultivate the ecosphere”). This is somehow reflecting the idea of Anthropocene in the field of the Bioeconomy.

The issues addressed above go in the direction of making the Bioeconomy a more and more complex system, not only involving the supply side but event more the integration of social and ecological system. For this reason, system-level organisational concepts are advocated to address the Bioeconomy. An area of attention is that of interpreting the Bioeconomy through the concept of socio-ecological systems [7, 20]. Even wider approaches are being proposed in order to enlarge the concept further, taking explicitly into account technology and value flows, e.g. as in the SETVIEW model [26].

## Bioeconomy and Circular Economy

D’Amato et al. [5] analyse the concepts of CE and Bioeconomy together also with the concept of Green Economy (GE) by reviewing around two thousand scientific articles published in the last 30 years.

Looking at the main keywords and topics, the results of the analysis indicate that the concept of the CE is very homogenous when compared with the other two. It strongly and monolithically focuses on resource efficiency, with mostly an industrial perspective, and with little or no references to the social dimension of the problem. It became popular in the 1990s, and it is roughly based on the idea of setting up an economic system with no or little effect on the environment, by advocating the design of products that minimise both input use and waste. The corollary is a re-organisation of the economy characterised by a strong inter-sectorial cooperation and coordination.

The Bioeconomy idea puts a stronger emphasis on the use of renewable resources as both input and energy source in the industrial production process. Corollary to this idea is, on one hand, the crucial role of agriculture and forestry in providing the raw input needed and, on the other hand, the role of research and innovation to develop the technology needed for the transformational processes. The authors highlight the need for the Bioeconomy to embed the idea of circularity, that, if absent, as in much of the literature, would lead to a business as usual scenario.

The GE concept synthetises much of the previous ones but adds to them a relatively stronger focus on nature-based solution (e.g. the role of ecosystem services) and on the social dimension of the problem. In this prospect, the GE seems to work as an umbrella terms, covering many of the aspects developed by the other two ones. On the contrary, the point of intersections between CE and Bioeconomy are rather limited, even though there is an increasing consensus on the need to reconcile them.

A striking result from the literature review by D’Amato et al. [5] is that while all the three concepts are interdisciplinary in their nature, economics as a discipline has largely ignored them, with some exceptions related to the GE. This is also the result of the paper by Gregorio et al. [14] where the great bulk of the papers on the economic aspect of the problem are related to management rather than economics. As a consequence, formal models of CE or Bioeconomy are rather scarce.

One of the few economics perspectives on the CE is provided by George et al. [13]. Their focus is on the optimal path of economic growth when production inputs can be both a polluting resource (such as oil) and a recycled material. Pollution is generated by the use of the polluting resource or by the stock of product that is not consumed or recycled. The model results follow in a rather mechanistic way. Given the assumptions of the model, the growth rate of consumption increases with the recycling ratio (the exogenously determined capacity of the system to recycle), and society will substitute the polluting with the recycled one as the cost of the former increases. Similarly to George et al. [13], García-Barragán et al. [12] develop an economic growth model but with the objective to measure to what extent a path is circular rather than linear. A key feature of their model is that consumer preferences depend on the functionality of the foods rather than of the materials that compose them. Production inputs are here as well both virgin and recycled materials and both entail externalities. The optimum entails that the stream of materials of both types is such that the future and marginal benefits are equal to the social marginal cost of recycling. Given these features, “the circular activity of the economy is defined as the difference between the optimal recycling activity and the optimal linear activity penalised by intolerance factors” [12]. In line with the findings of the literature review by D’Amato et al. [5], the formal models addressing the CE revolve around the concept of recycling. In this prospect, an integration with the Bioeconomy perspective would be useful in adding two dimensions to the problem of circularity: (1) the nature of the raw material (whether is renewable or not) and (2) the *costly* research and development activities needed to develop the technologies required.

Moving to the Bioeconomy, Zilberman et al. [31] provide a simple and illustrative economic treatment of the idea. The authors sketch the main issues related to the share of renewable and non-renewable resources, taking energy production as an example. The use of non-renewable resources is characterised by high investment costs and very low variable costs that however increase over time as the resource becomes scarcer and scarcer. A technology that relies on renewable resources entails instead even higher investment costs, and low variable costs that might even decrease over time as the technology develops. As long as the exploitation of the resource does not exceed the rate of the regeneration, its use in the production activities can be sustained over time. More likely the two systems coexist, and the optimal share of the use of the two resources is given by the equalisation of the demand with their social marginal costs. The model depicted is rather simple and leaves out more questions than they solve.

The first point that must be addressed is the fact that while biological resources are renewable, they need land to be harvested and cultivated, and land is a finite resource. In this prospect, while biological resources can be sustained indefinitely, the marginal cost of using land might be increasing, as land with lower and lower quality is put into production. This raises another issue (raised by Zilberman et al. [31] too): the use of land for agriculture is in competition with ecosystem services and habitat, and the use of land for producing industrial inputs is in competition with food production [30].

Moreover, coordination among the sectors is not addressed while it seems to be a precondition for the emergence of the Bioeconomy [18]. In this prospect, however, the CE focus on recycling might be of help. Indeed, if the focus of Bioeconomy technologies revolves around the use of waste of food production, the aforementioned trade-offs are likely to be reduced, even though not to disappear. A cross-cutting issue is the role of research and development. The technology needed by the Bioeconomy revolution need to be developed through research. Research, however, is not a free lunch. From the economic point of view, an issue to be further deepened is the role of research funding for the development Bioeconomy technology and its relative added value to other mechanisms such as taxes in the reduction of negative externalities.

Moving from the theoretical analysis to the actions taken by actual firms, the need for the Bioeconomy to embed the notion of circularity at the core of the CE concept is highlighted by, e.g. Lokesh et al. [18]. In the framework of the Bioeconomy, however, some value chains cannot embrace circularity, as biomass used for energy delivery for example imply a dead-end. Lokesh et al. [18] map the potential value chains for which circularity can become a promising goal. D’Amato et al. [6] analyse to what extent companies involved in land use intensive sectors embrace the concepts aforementioned and use them to communicate their sustainability efforts. CE is by far the most dominant concepts across sectors and across continents. The Bioeconomy is the least used idea, and it is predominantly employed by firms located in Europe and in the forest sector. The notion of Bioeconomy and circularity entails substantial changes in the business model of the firms. Business models have been reviewed by Salvador et al. [21], who highlight several issues and hint at how to develop them for firms working in the circular-bioeconomy. Among the others, the development of new technologies is not itself a motive for success. This is especially true for the sector at stake, where the product itself is going to substitute existing ones, and thus it is difficult, and needed, to properly communicate the novelty of the product. Moreover, logistics is relatively more important for the circular Bioeconomy than for other traditional sectors, as transportation costs might represent a big share of total production costs. An attention to local markets, especially with respect to the input delivery, is of paramount importance.

Additional considerations of interest may be attached to the peculiarity of circularity related to biological resources. First, the fact that the biomass, sooner or later is degraded, implies that taking a system and time span large enough, the Bioeconomy cannot be completely circular. Second, circularity is connected to input availability and circularity (e.g. for fertilisers). Third, it is important the separation, but also the interplay, between circularity of biological and non-biological resources.

## Towards a Framework for Circular Bioeconomy Organisation and Evaluation in the Post-COVID Era

There are several consequences of the trends illustrated above. We point here to three main relevant issues:

- The need to consider circular processes in the appropriate way, including related economic aspects
- The need to cast circularity in the increased complexity and interconnection of downstream and upstream markets
- The need for new forms of interaction between demand and supply

These topics have taken increasing importance over time for bio-based systems. However, the outbreak of the COVID-19 pandemics has boosted their importance and the need for attention. In particular, the COVID-19 outbreak has pointed attention to the need for flexible and resilient systems, able to respond to totally unexpected situations. This goes beyond what has actually happened during the COVID outbreak, as attention now is not so much to the possible replication of this pandemic, but rather to the idea that completely new shocks may occur, possibly taking different (and unknown) forms and systems need to be ready for this uncertainty.

The need to consider circular processes through a proper economic analysis relates to the issue of addressing appropriately the economic aspects of circularity in the valuation. The main drawbacks are the lack of consideration of the distinction between renewable and non-renewable resources and the poor consideration of research and the dynamics of the optimal share of non-renewable resources. This is connected to management issues, such as the fact that production cost accounting with recycling requires appropriate approaches. The key background issue relates to the economic problem of taking into account or even evaluate the optimal level of recycling or of circularity in a system [17, 28].

**Fig. 3:**
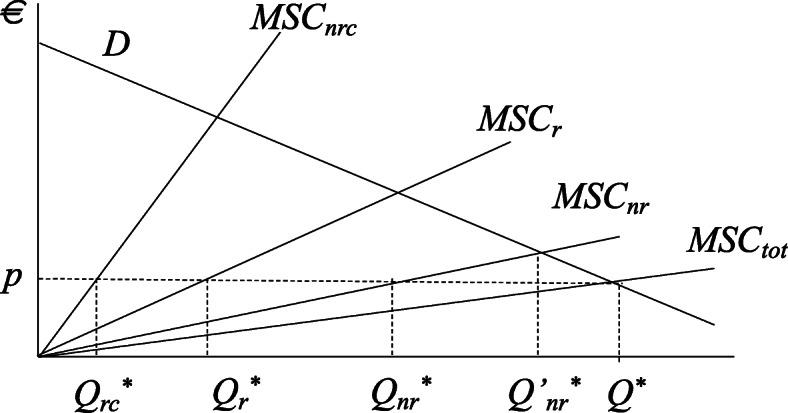
Optimal level of circularity with renewable, non-renewable and non-renewable recycled resources

Figure 3 builds upon Zilberman et al. [31] and illustrates the economic forces at stake in determining the optimal amount of non-renewable resources, renewable ones and recycled resources. Assume that the demand of input does not distinguish among these typology of resources. On the other hand, each of the resource is delivered at different marginal costs, entailing different technologies. The figure depicts the social marginal costs for each of the resource typologies; such marginal costs include both the private one and the externalities associated with them. The optimal quantity is provided at the point where the demand is equal to the total marginal social costs (*MSC*_*tot*_) leading to a clearing price of *p*. The share of each of the resource typologies is found by intercrossing the price *p* with the respective MSC lines.

Note that if the non-renewable resource was the only one available, the total social costs would be given by *MSC*_*nr*_, and the total quantity provided would be *Q’*_nr_*. The introduction of the new technologies, recycling and the Bioeconomy has two effects. The first one is to increase the total supply of the resource, causing a reduction in the clearing price. The second one is a substantial reduction in the use of the non-renewable resource that is substituted with the other two. Simple comparative statics shows that a reduction in the marginal costs of any of the resource delivery has similar effects: an increase in the total supply, a reduction in the price of the resources, and an increase in the use of the resource for which the marginal costs has reduced and a parallel reduction in the use of the other resources.

Important pieces of information are however missing in this simple representation. Technology adoption entails (often high) fixed costs that, while do not affect input use at the margin, affect the adoption of the technology as a whole.

Assuming that higher circularity has a higher marginal cost (higher marginal costs of recycling technology and lower marginal environmental cost due to decrease pressure on the environment), it is likely that there will be a non-trivial issue in understanding what level of circularity is optimal and how it evolves over time. This needs to be further investigated upfront, in order to support meaningful (from an economic point view) transition processes. It however requires the consideration of multiple information and the evaluation of trustable marginal supply curve per technology. This is clearly a difficult area for economic valuation, but it is also the area likely allowing the higher benefit for the analysis of circular Bioeconomy systems.

All these problems were born to solve the problem of sustainability and of environmental degradation. But the technologies to solve this issues (recycling and Bioeconomy) need research that is not a free lunch. One could ask, for example, what is better: taxing polluting activities or investing in research, or both (with may be connected use of funding)?

As a second point of attention, the complexification of the system and the increasing number of separability points need to be considered together with the concept of circularity. In other words, circularity does not mean just re-using products prior to disposal, but exploiting the possibilities offered by the system in terms of connection among chains, or, where suitable, the degradation of the organic material to the most simple components in such a way to allow for the maximum usability in different options. This is also an aspect that allow higher level of flexibility in principle and hence potentially contribute to a system needing resilience. Together with resilience, however, it can bring also potential problems. One of them is the increase of interconnections among markets of different products that can increase volatility of prices, or unpredictability/volatility of economic performance for individual firms, due to interlinkages of shocks.

Summing up the two points above, we can imagine expanding the concept of Figure 3 to a mix of tens or hundreds of potential sources of supply for the same product, interacting among each other, with a degree of flexibility and complexity exacerbated by circularity.

As a result of the above, top-down organisational solutions become less and less credible and in turn new forms of governance gain importance. This is also relevant for economic research and for the kind of contribution economics and management can give to the process. One of the most evident changes looking at the current trends in, e.g. research funding, is the move from cost analysis to business models. Far from being incidental, this move reflects the understanding that cost estimation is quickly obsolete and may require a number of assumptions. In addition, costs tend to look at the issue of drop-in products (product with same features from a new process), rather than looking at exploiting the potential of new products. Business models, on the contrary, look explicitly at the way to provide value for clients and society. On the other hand, business models are also difficult to study as they acknowledge the evolutions of contexts and companies, the potential for success and failure and the role of creativity and entrepreneurship, letting alone the role of context characteristics, heterogeneity and local adaptation.

Taking into account the need of flexibility and resilience in the post-COVID era, these trends offer clearly an opportunity to achieve a bioeconomy system able to face unexpected events and to react to shocks. However, flexibility may be costly either in terms of volatility or coordination costs and needs hence to be governed thorough appropriate processes.

In this direction, it is apparent how evaluation will become more important, but also more complex and difficult, due to the need to account for dynamic processes and a larger variety of interconnected markets. In addition, it will become more uncertain. As a consequence of the combination of high potential and high uncertainty, evaluation will need to become an inherent aspect of decision-making processes, more than in the past, but, at the same time, with a higher awareness of difficulties and limitations also by users of the evaluation. This entails the need to address evaluation having in mind a broader context than in the past, both in the sense of geographical scope and time horizon, but also in terms of the variety of the economic sectors considered.

While this is challenging, these trends also bring certainly opportunities linked to the growing demand for evaluation and valuation, as well as demands for methodological innovation. The concept of business model already goes in the direction of exploring value creation with a view at the interconnection between demand and supply. Key to this innovation will be the combination of new evaluation needs, higher computational needs and new data availability landscape brought by the process of digitalisation.

An aspect of this is also a renewed attention to the mechanisms related to the creation of value, with different branches of the literature pointing in turn to either the new promissory economies linked to innovation and financial markets [1, 2] or to new concepts of values due to the use of living organisms, such as the biovalue [3].

## Discussion and Final Remarks

The development of a CBE is certainly bringing several challenges, still to some extent hidden by the partial understating of the current social and economic transition. In this paper we have attempted to provide a framework to address the issue of Bioeconomy and circularity in the post-COVID era, building on a classical comparative statics framework. We show how circularity contributes to the increase of connections among markets and how this interacts with separability in boosting the complexification of the Bioeconomy system. It also contributes, at the same time, to flexibility and uncertainty. As a result, moving to CBE also requires attention to appropriate economic organisation concepts and evaluation procedures, allowing to address novel concepts such as flexibility, resilience and complex systems rather than mere advocacy and description of value chains. In this direction, it is also apparent how evaluation will become more important, but also more complex and difficult, due to the need to account for dynamic processes and a larger variety of interconnected markets.

These issues are particularly relevant for the primary sector, because the increased flexibility and variety of processing approaches also reflect in higher substitutability among biomass sources, hence high competition and volatility of markets, as well as the need of providing clear anchoring between territory and chains based on socio-economic structures rather than physical constraints.

Finally this process is probably further promoted by the effects of the pandemics, calling for an increase in flexibility, resilience and preparedness to future (unknown) shocks. In the context of the pandemics, however, it is likely that the potential trade-offs between flexibility and fairness will be more balanced, due to the high social attention for the topic.

The main limitations of this paper rest in the lack of empirical application and in the simplified qualitative elaboration on alternative organisational pathways. On the other hand, these limitation hint at pathways for future research and improved approach to policy and actors support. Two clear directions for further research are related to: (a) the investigation of system-level organisational models, properly accounting for value web structures, and (b) evolution of business models research in the direction of better incorporating the interaction with these networks.

## Data Availability

Not applicable

## Data Availability

Not applicable: the paper is a review not using specific data.
